# EDD, a Ubiquitin-protein Ligase of the N-end Rule Pathway, Associates with Spindle Assembly Checkpoint Components and Regulates the Mitotic Response to Nocodazole*

**DOI:** 10.1074/jbc.M114.625673

**Published:** 2015-04-01

**Authors:** Flavia Scialpi, David Mellis, Mark Ditzel

**Affiliations:** From the MRC Institute of Genetics and Molecular Medicine, University of Edinburgh, Edinburgh EH4 2XR, Scotland, United Kingdom

**Keywords:** cell cycle, checkpoint control, chromosomes, E3 ubiquitin ligase, mitosis, BUB3, BUBR1, CDC20, EDD, SAC

## Abstract

In this work, we identify physical and genetic interactions that implicate E3 identified by differential display (EDD) in promoting spindle assembly checkpoint (SAC) function. During mitosis, the SAC initiates a mitotic checkpoint in response to chromosomes with kinetochores unattached to spindle pole microtubules. Similar to *Budding uninhibited by benzimidazoles-related 1 (BUBR1*) siRNA, a *bona fide* SAC component, *EDD* siRNA abrogated G_2_/M accumulation in response to the mitotic destabilizing agent nocodazole. Furthermore, *EDD* siRNA reduced mitotic cell viability and, in nocodazole-treated cells, increased expression of the promitotic progression protein cell division cycle 20 (CDC20). Copurification studies also identified physical interactions with CDC20, BUBR1, and other components of the SAC. Taken together, these observations highlight the potential role of EDD in regulating mitotic progression and the cellular response to perturbed mitosis.

## Teaser

**Background:** Mitotic progression is regulated by the spindle assembly checkpoint (SAC) to prevent aneuploidy and chromosome damage.

**Results:** EDD binds to various SAC components, governs the expression levels of key mitosis-associated proteins, and mediates the response to the mitotic spindle poison nocodazole.

**Conclusion:** EDD contributes to the ability of the SAC to mediate checkpoint arrest.

**Significance:** EDD may act to maintain genomic integrity.

## Introduction

EDD^2^ (E3 identified by differential display), also known as UBR5 or hHYD, is an evolutionarily conserved homologous to E6-AP carboxyl terminus bearing E3 ubiquitin-protein ligase of the N-rule pathway (1) and homolog of *Drosophila* hyperplastic discs (Hyd), a *Drosophila* tumor suppressor involved in controlling tissue growth and differentiation (2–4). Evidence to support a conserved role for the human homolog in tumorigenesis comes from its high mutational frequency in diverse cancers (COSMIC, Wellcome Trust Sanger Institute), with a particular high incidence in breast (5) and mantle cell carcinoma (6).

Although implicated in DNA damage-mediated control of cell cycle progression (7–10), EDD has not yet been associated with SAC-associated regulation of mitosis. The SAC is a multiprotein complex that comprises mitotic arrest deficient 2 (MAD2), Bub1-related protein kinase (BUBR1), and budding uninhibited by benzimidazoles 3 (BUB3). Acting together, they provide an essential mitotic checkpoint that maintains chromosomal integrity, ensures correct chromosome separation, and prevents aneuploidy (11). Triggered by kinetochores unattached to the mitotic spindle, activation of the SAC delays metaphase-anaphase transition to allow Aurora B kinase-mediated error correction mechanisms to promote kinetochore attachment (12–14). Mechanistically, the SAC achieves the temporal delay in anaphase progression by inhibiting cell division cycle 20 (CDC20), a substrate specificity factor for the multisubunit E3 APC/C (11). SAC-associated CDC20, collectively referred to as the mitotic checkpoint complex (MCC), is unable to promote APC-mediated degradation of metaphase-to-anaphase inhibiting proteins such as Cyclin B and Securin (11). Here we identify physical interactions between EDD, CDC20, and components of the SAC and reveal the potential role of EDD promoting mitotic arrest in response to Noc.

## EXPERIMENTAL PROCEDURES

### 

#### 

##### Plasmids, siRNA Oligos, and Transfections

The *EDD*, *BUB3*, and *BUBR1* coding sequences were amplified by PCR from HeLa total cDNA and cloned into a modified pcDNA5/FRT (Life Technologies) containing an amino-terminal 2×HA/2×Strep (HS) or V5/FLAG (VF) epitope tags. Plasmid transfections were performed using Effectene (Qiagen) according to the protocol of the manufacturer or with the *N,N*-bis[2-hydroxyethyl]-2-aminoethanesulfonic acid/CaCl_2_ method (Life Technologies). *EDD* and *BUBR1* were silenced using Lipofectamine RNAiMax (Life Technologies) with the following oligos: *siEDD1*, 5′-CTCGTCTTGATCTACTTTATC-3′; siEDD2, 5′-GUGUAUCAGUUUGCUUUCCAA-3′; scramble control, 5′-GAACCAAAGUAGCAUAUAACU-3′; and *siBUBR1*, 5′-CAUAUUCAAAUGCCCGUU-3′.

##### Cell Culture and Nocodazole Treatment

An inducible HEK293 cell line expressing HS-EDD was created using FLP-In^TM^ HEK293 T-REx (Life Technologies). HEK293 and HeLa cells were grown at 37 °C/5% CO_2_ in high-glucose DMEM (Sigma) supplemented with 10% FBS (Life Technologies) and 4 mm l-glutamine (Sigma). HeLa cells were arrested in mitosis by treating them with either 50 ng/ml nocodazole or 10 nm Taxol for 18 h. Mitotic cells were harvested by a physical “shake-off.”

##### Pulldown Assays, Immunoprecipitation, Immunoblotting, and Mass Spectrometric Identification

Transfected HS-EDD HEK293 were induced with 1 mg/ml doxycycline for 4 h before harvesting. Cells were then washed once in ice-cold PBS and lysed with Triton lysis buffer (50 mm Tris (pH 7.5), 100 mm NaCl, 2 mm EDTA, 1% Triton X-100, 1× Roche protease inhibitor mixture, and 1× Roche phosphatase inhibitor mixture). The lysates were clarified by centrifugation at 13,000 rpm for 15 min at 4 °C in a benchtop rotor. V5/FLAG-BUB3 was pulled down using FLAG-M2 beads (Sigma) for 1 h at 4 °C. Beads were washed three times with lysis buffer, and protein complexes were eluted with 1 bead volume of 1× Laemmli sample buffer with 5% β-mercaptoethanol. To immunoprecipitate endogenous BUB3 and BUBR1, cells were lysed as above and precleared using appropriate isotype IgG-conjugated beads (Cell Signaling Technology) for 45 min at 4 °C and then incubated with the appropriate antibody overnight at 4 °C. Samples were then incubated with the appropriate IgG F(ab')2-conjugated beads (Cell Signaling Technology) for 30 min at 4 °C, spun down, and eluted with 1 bead volume of 1× Laemmli sample buffer. Precast NuPAGE® BisTris gradient gels (Life Technologies) were used for SDS-PAGE, run with MOPS buffer, and semidry-transferred onto PVDF prior to Western blot analysis. Antibodies used were as follows: mouse FLAG M2 (Sigma, catalog no. F3165); rabbit FLAG M2 (Sigma, catalog no. F7425); goat EDD M19 (Santa Cruz Biotechnology, catalog no. sc-9561); mouse CDC27/APC3 (Abcam, catalog no. ab10538); rabbit CDC20 (Santa Cruz Biotechnology, catalog no. sc-8358); mouse p21 (Santa Cruz Biotechnology, catalog no. sc-6246); mouse BUB3 (catalog no. BD 611730); rabbit BUBR1 (Bethyl, catalog no. A300-386A); sheep BUBR1 (a gift from Dr. Stephen Taylor, University of Manchester); and anti-rabbit, anti-mouse, and sheep IgG HRP-linked secondary antibody (Cell Signaling Technology).

For the mass spectrometry studies, lysates from four 15-cm plates of HS-EDD or HS tag HEK293 cells were tandem affinity-purified by HA and Streptactin-based affinity resins (Invitrogen). Purified proteins were separated into high and low molecular weights by SDS-PAGE, in-gel digested using trypsin, and fractionated using strong cation exchange. Fractions were desalted and analyzed using LC-MS on a LTQ-Orbitrap (Thermo Fisher Scientific) coupled to HPLC. The MS data were analyzed using MaxQuant, and proteins were identified by searching MS and MS/MS data using the MASCOT search engine.

##### Live Imaging

HeLa cells expressing red fluorescent protein-tubulin (RFP-tubulin) and GFP-histone H2B (15) were silenced with siRNAs using Lipofectamine RNAiMAx (Life Technologies). 48 h after transfection, cells were placed in complete DMEM without phenol red in a temperature- and CO_2_-controlled incubation chamber (Solent Scientific Ltd), and images were acquired every 10 min using a Nikon Ti Eclipse inverted microscope, a Nikon ×10 Plan Fluor 0.3 numerical aperture Ph1 lens, and Nikon fluorescence filter sets for GFP and RFP (Nikon UK Ltd). Image acquisition was performed using a Photometrics Coolsnap HQ2 charge-coupled device camera (Photometrics Ltd) and Nikon Nis-Elements advanced research software (Nikon Instruments Europe). A combination of brightfield, RFP-tubulin, and GFP-H2B images was used to determine cells entering mitosis, progressing, exiting mitosis, and undergoing cell death. Using these criteria, we were able to determine the total number of cells attempting mitosis over a 10-h time course separated into 10-min time frames. Cells termed “successfully completing mitosis” entered, progressed, and exited mitosis without exhibiting any defects. Cells initiating mitosis and either exhibiting cell death within or shortly after mitosis or failing to complete cytokinesis were deemed to be cells attempting but failing to successfully complete mitosis.

##### FACS Analysis

For FACS analysis, silenced HeLa cells were washed once with PBS, fixed with ice cold 70% EtOH, and stained with DAPI (1:2500). Anti-**γ**H2AX antibody (catalog no. 05-636, Millipore) was used for DNA damage studies and analyzed according to Huang and Darzynkiewicz (16) without cell cycle phase determination. Samples were analyzed using a BD FACS Aria III and FACSDiva software (BD Biosciences).

##### Immunofluorescence

HeLa cells seeded on coverslips were washed with PBS and fixed in 4% paraformaldehyde:PBS for 10 min at room temperature. After washing with PBS, cells were permeabilized using 0.25% Triton X-100:PBS for 5 min, blocked in 10% BSA:PBS for 30 min, and incubated with the appropriate primary antibodies at 4 °C overnight. Antibodies used were as follows: rabbit EDD (Bethyl, catalog no. IHC-00025); mouse BUB3 (catalog no. BD 611730); and sheep BUBR1 (a gift from Dr. Taylor, University of Manchester). After washing with PBS, cells were incubated for 30 min at room temperature with the appropriate fluorescently conjugated secondary antibody (488-sheep A11015, 488-mouse A21202, and 594-rabbit A21207; Life Technologies) and mounted in Vectashield containing DAPI (Vector Laboratories, Inc.). All antibodies were diluted in blocking solution. Images were captured on a Zeiss Axioplan II fluorescence microscope and analyzed using the Volocity software (PerkinElmer Life Sciences).

##### Graphing, Statistical, and Image Processing Software

Microsoft Excel and GraphPad Prism were used to produce graphs and carry out statistical analyses. Adobe Photoshop and Illustrator were used to manipulate the images and lay out the figures, respectively.

## RESULTS

### 

#### 

##### EDD Complexes with the SAC Components BUB3 and BUBR1

**FIGURE 1.**
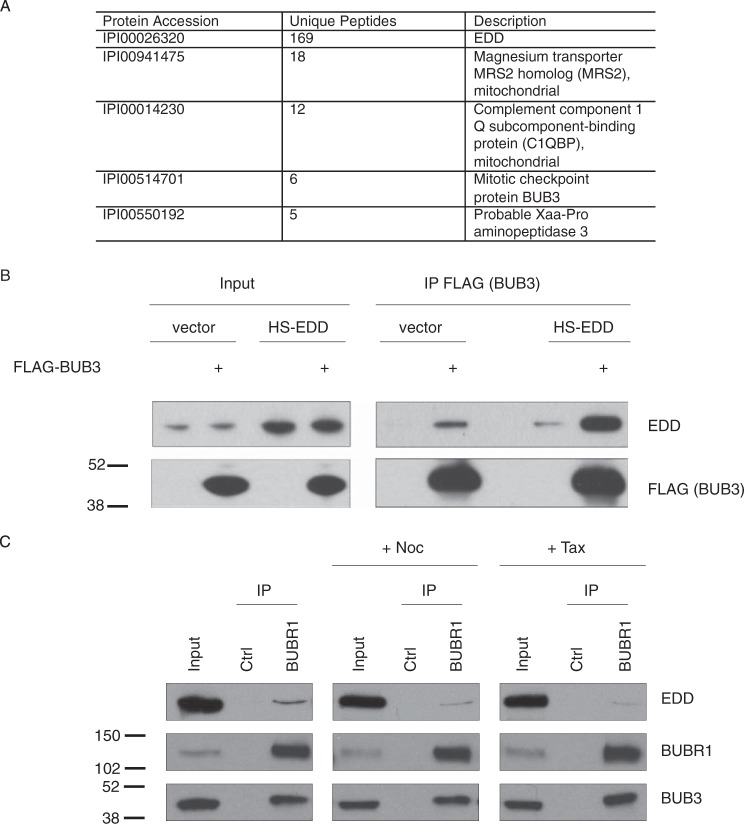
**EDD coimmunoprecipitates with BUB3 and BUBR1**. *A*, MS/MS-based identification of HS-EDD copurifying proteins from HEK293 cells (*n* = 3). Any HS-EDD copurifying proteins identified in the HS-only sample were removed for the HS-EDD potential interactor list. The five top hits are shown and include the EDD bait, two mitochondrial proteins (MRS2 and C1QBP), BUB3, and Xaa-Pro aminopeptidase 3. *B* and *C*, HEK293 (*B*) and HeLa cells (*C*) were either transfected with the indicated constructs (*B*) or treated with either Noc or Tax (*C*) prior to IP with the indicated antibodies or IgG controls (*Ctrl*). Lysates (*Input*) and coimmunoprecipitates were analyzed by SDS-PAGE and Western blotting with the indicated antibodies. Quantification of immunoprecipitated EDD in *C* indicated a 69% and 81% reduction upon nocodazole and Taxol treatment, respectively. Images are representative of two independent experiments. Molecular weight standards are indicated. Note that EDD is 309 kDa and runs well above the high molecular weight marker (250 kDa).

To identify new interactors of the 309-kDa EDD protein, we created a stable doxycycline-inducible HS-tagged EDD (HS-EDD) HEK293 cell line using site-specific recombinase FLP-mediated recombination. Mass spectrometry of purified HS-EDD-complexed proteins revealed the mitotic checkpoint protein BUB3 to be a potential novel interactor (Fig. 1*A*). Encouragingly, we also identified Xaa-Pro aminopeptidase 3, a previously identified EDD interactor (17). To verify the BUB3 interaction, we performed co-IP studies in asynchronous HEK293 cells, which confirmed an interaction between exogenous FLAG-BUB3 and both endogenous EDD and exogenous HS-EDD (Fig. 1*B*). Because of the role of BUB3 in the SAC, we addressed whether EDD could also bind to BUBR1. IP of endogenous BUBR1 from asynchronous HeLa cells co-IPd both endogenous EDD and BUB3 (Fig. 1*C*, *left panel*). To examine the interaction profile of EDD in mitotic cells with an activated SAC, we performed BUBR1 co-IP studies with HeLa cells treated with the microtubule poison agent Noc (18) or the microtubule-stabilizing agent Taxol (Tax) (19) (Fig. 1*C*, *center* and *right panels*, respectively). In comparison with untreated asynchronous cells, both spindle poisons resulted in a small, but reproducible, reduction in co-IPd EDD. However, there was no reduction in the interaction between BUBR1 and BUB3, suggesting that the interaction of EDD with BUBR1 may preferentially occur either outside of metaphase and/or in the absence of spindle poisons.

##### EDD Complexes with MCC- and APC/C-associated Factor CDC20

**FIGURE 2.**
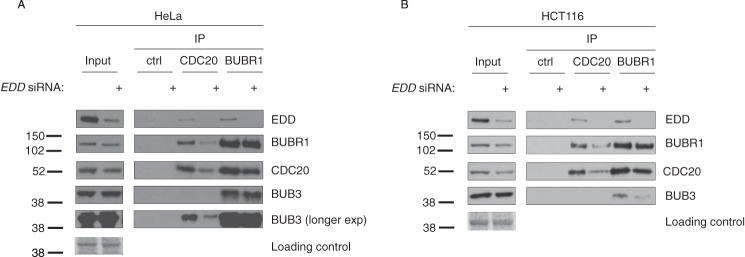
**EDD coimmunoprecipitates with SAC- and APC-associated components.** *A* and *B*, HeLa (*A*) and HCT116 (*B*) cells were treated with either *EDD* or scramble control siRNAs. Following siRNA treatment, lysates were immunoprecipitated with CDC20, BUBR1, or IgG control (*Ctrl*) antibodies, and coimmunoprecipitating proteins were analyzed by SDS-PAGE and Western blotting with the indicated antibodies. Ponceau Red-stained membranes are included as a loading control for the lysate inputs. Images are representative of two independent experiments.

The ability of EDD to bind BUBR1 and BUB3 suggested that it might influence the formation or stability of the SAC and/or the CDC20-containing MCC. To address this, we carried out co-IP studies in two different cell lines (Fig. 2). Using asynchronous HeLa cells, we first addressed whether *EDD* siRNA would affect the interaction of BUBR1 with endogenous CDC20 and BUB3 (Fig. 2*A*). Comparison of BUBR1 IPs from scrambled siRNA-treated (control) and *EDD* siRNA-treated HeLa cells revealed no differences in the amount of coimmunoprecipitated CDC20 or BUB3. Of note, *EDD* siRNA did not affect BUBR1 or BUB3 expression levels in the input lysates. Consistently, *EDD* siRNA in both cell lines resulted in a small decrease in the CDC20 inputs that accompanied a decrease the amount of IPd CDC20. Concurrently, a similar reduction was also observed with coimmunoprecipitated BUBR1 and BUB3. Overall, the effects observed in HeLa cells were very similar to those observed in HCT116 cells (Fig. 2*B*), with *EDD* siRNA reducing CDC20 expression in the lysate. In summary, *EDD* siRNA appeared to affect CDC20, but not BUBR1 complexes.

##### The Subcellular Localization of EDD Changes through Mitosis and Colocalizes with BUB3

**FIGURE 3.**
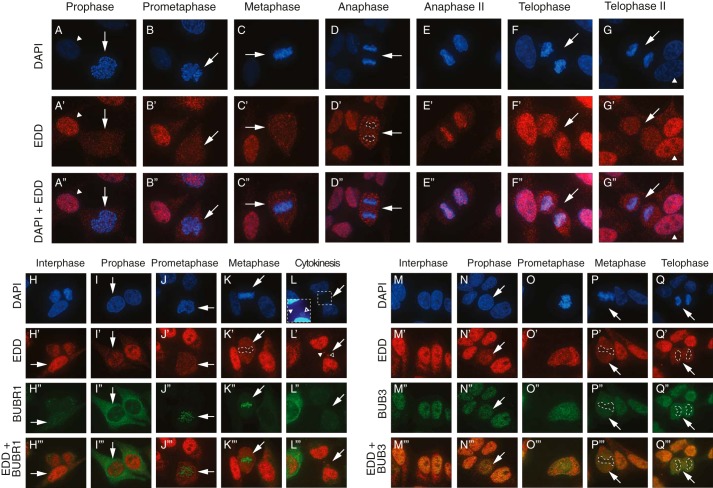
**EDD, BUBR1, and BUB3 immunofluorescence analysis during mitosis.** *A–Q”'*, asynchronous HeLa cells were fixed, stained with DAPI, and processed for immunofluorescence with the indicated primary antibodies. Interphase and distinct mitotic phase cells were determined by DAPI-based DNA staining, with the relevant cell of interest marked by an *arrow*. Overlays of DAPI + EDD (*A”–G”*), EDD + BUBR1 (*H”'–L”'*), and EDD + BUB3 (*M”'–Q”'*) are also shown. *Dashed lines* indicate areas of low colocalization between the protein of interest (low signal) and DAPI staining (high signal). In *A–G”*, the EDD images are representative of at least >70% of the cells analyzed in each mitotic phase (prophase, 26 of 33; prometaphase, 7 of 7; metaphase, 26 of 31; anaphase, 4 of 4; anaphase II, 3 of 3; telophase, 14 of 14; telophase II, 5 of 7).

Because of the ability of EDD to bind SAC components, we wished to establish the mitotic subcellular localization of EDD and its ability to colocalize with chromosomes, BUB3 and BUBR1. Previous reports have revealed EDD to be a nuclear protein (20–22). However, its specific expression through mitosis was not determined. Using immunofluorescence, we revealed EDD to be present in small puncta throughout the cell in prophase, prometaphase, and metaphase (Fig. 3, *A'–C'*, *arrows*). During anaphase, EDD signals formed larger puncta that appeared to be excluded from the chromosomes (Fig. 3*D*', *dashed lines*). Intriguingly, during late anaphase (anaphase II), the pattern observed in early anaphase was reversed, with EDD puncta almost exclusively colocalizing with the chromosomes (Fig. 3*E”*). As telophase progressed, EDD signal localization became more diffuse and less concentrated on chromosomes (Fig. 3, *F”* and *G”*, *arrows*). Non-mitotic interphase cells demonstrated strong nuclear EDD staining punctuated with weak staining within regions of low DAPI intensity (presumed nucleoli, Fig. 3, *G–G”*, *arrowheads*). In comparison with telophase and interphase cells, EDD signal intensity was reduced during prophase to anaphase (Fig. 3*A*', compare interphase (*arrowhead*) and prophase (*arrow*) cells).

In light of the physical interactions of EDD with BUBR1 and BUB3, we wished to determine its ability to colocalize with them (Fig. 3, *H–L”'* and *M–Q”'*). Throughout mitosis, BUB3 (Fig. 3, *N–Q”'*), but not BUBR1 (Fig. 3, *I–K”'*), showed very similar expression patterns as EDD. In interphase, the nuclear localization of EDD overlapped with BUBR1 cell-wide- and BUB3 nuclear expression patterns (Fig. 3, *H”'* and M”', respectively). During prophase, EDD signals failed to significantly overlap with the predominantly perinuclear and cytoplasmic staining of BUBR1 (Fig. 3*I”'*). Similarly, during prometaphase and metaphase, BUBR1 and EDD puncta failed to significantly colocalize, with BUBR1 signals preferentially colocalizing with DAPI (Fig. 3*K”*), indicative of its association with kinetochores (23). The region of intense BUBR1 signal also corresponded with a region of low EDD signal (Fig. 3*K'*, *dashed line*). Daughter cells undergoing cytokinesis revealed EDD to be associated with nuclei, potential micronuclei (Fig. 3, *L*, *inset*, and *L'*, *inset*, *open arrowhead*), and the cytokinetic neck/junction, an area that lacked a DAPI signal (Fig. 3, *L*, *inset*, and *L'*, *closed arrowheads*).

In contrast with BUBR1, BUB3 was nuclearly localized in interphase (Fig. 3*M”*) and evenly distributed across the cell during prometaphase (Fig. 3*O”*). As with EDD, BUB3 appeared to be excluded from the DNA during metaphase and telophase (Fig. 3, *P”* and *Q”*, respectively, *dashed lines*). Therefore, EDD exhibited strong colocalization with BUB3 throughout mitosis and weaker colocalization with BUBR1 during interphase and prophase. These observations may also help to explain why less EDD was coimmunoprecipitated with BUBR1 upon Noc- or Tax-mediated enrichment of metaphase cells (Fig. 1*C*). Taken together, these data suggest that the physical interaction between EDD and BUBR1, but not BUB3, may be regulated by subcellular compartmentalization/sequestration.

##### EDD siRNA Abrogates G_2_/M Arrest in Response to Noc Treatment

**FIGURE 4.**
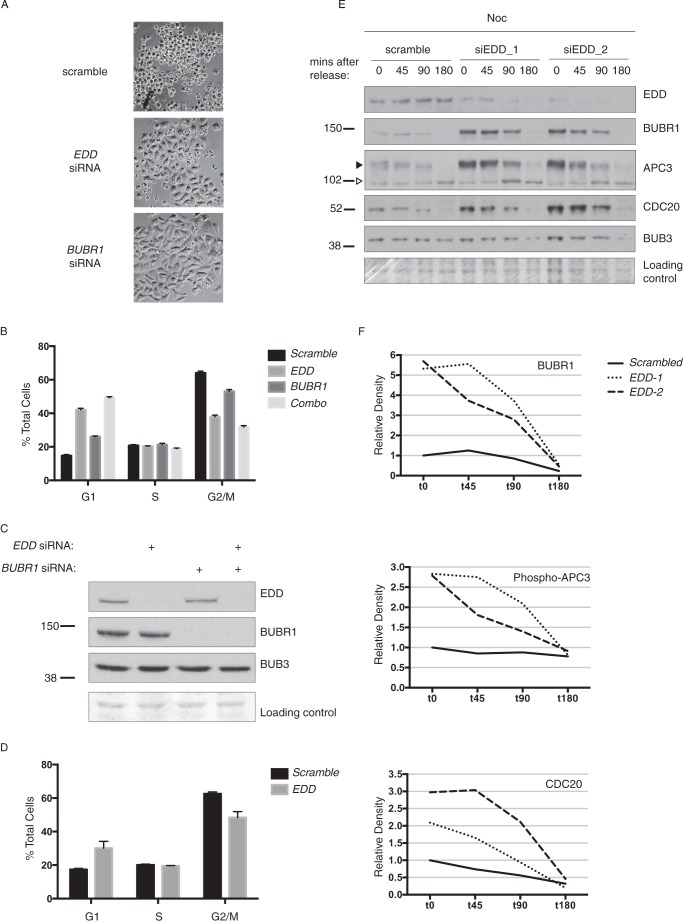
**EDD siRNA abrogates nocodazole-mediated G_2_/M accumulation and increases expression of CDC20, phospho-APC3, and BUBR1.** HeLa cells were treated with the indicated siRNAs, treated with nocodazole (*A–C*, *E*, and *F*) or Taxol (*D*) for 18 h, released, and imaged by brightfield microscopy (*A*) and analyzed by propidium iodide staining and FACS analysis (*B* and *D*, respectively). siRNA efficiency (*C*) and siRNA-treated cells released from Noc for the indicated times post-release (*E*) were analyzed by SDS-PAGE and Western blotting with the indicated antibodies. *F*, density quantification of bands in *E*, with the values expressed relative to scrambled siRNA t0 values. The data in *B* and *D* were analyzed using one-way analysis of variance and Tukey's multiple comparison test, which revealed all G_1_ or G_2_/M comparisons to be significantly different.

Because of the ability of EDD to associate with SAC/MCC components, we hypothesized that it may also govern a mitosis-associated response to Noc treatment. To address this, HeLa cells were first treated with scrambled, *EDD*, or *BUBR1* siRNAs, followed by Noc treatment and FACS analysis. Examination of the *EDD*- and *BUBR1* siRNA-treated cells revealed a visible decrease in the appearance of rounded cells in comparison with the scrambled control (Fig. 4*A*). In comparison with the scrambled siRNA-treated cells, FACS analysis revealed the expected *BUBR1* siRNA-mediated reduction in the number of G_2_/M cells (Fig. 4*B*), reflecting the reported impairment of SAC function and an inability to arrest in M phase (23). Similarly, *BUBR1* siRNA cells showed an increased G_1_ cell population, which was presumably due to a combination of increased cellular flux of G_2_/M cells into G_1_ and/or activation of the G_1_ checkpoint as a consequence of the mitosis-associated chromosomal defects and DNA damage (24). A similar overall cell cycle profile was also observed in *EDD* siRNA cells. However, the effects on the G_1_ and G_2_/M cell populations were more pronounced (Fig. 4*B*). Importantly, Western blot analysis of the siRNA-treated cells revealed the expected siRNAi-mediated knockdown of their appropriate targets (Fig. 4*C*). Therefore, both *BUBR1* and *EDD* siRNA cells exhibited decreased G_2_/M and increased G_1_ cell populations in response to Noc treatment. Interestingly, combined *EDD* siRNA and *BUBR1* siRNA resulted in a more pronounced cell cycle profile than that of *EDD* siRNA alone. This additive effect between *EDD* and *BUBR1* siRNA, combined with the physical association between the two proteins, suggests that they may both function to normally promote a G_2_/M arrest and suppress G_1_ arrest in response to Noc. *EDD* siRNA treatment also caused a similar cell cycle profile in response to Taxol treatment (Fig. 4*D*).

##### EDD Represses BUBR1, APC3, and CDC20 Expression in Mitosis

Next, to gain insight into the potential mechanism underlying *EDD* siRNA abrogation of the Noc-mediated G_2_/M accumulation, we examined the mitotic expression of key mitotic regulators. HeLa cells were treated with Noc, and mitotic cells were shaken off, replated, and then allowed to progress through mitosis in Noc-free medium. Cells were harvested at various time points following Noc release and analyzed by Western blotting (Fig. 4*E*). In scramble siRNA-treated cells, BUBR1 and CDC20 levels decreased steadily as mitosis progressed, as did the phosphorylated form of the APC subunit APC3, a marker of APC activity (25). In contrast, BUB3 and EDD expression levels remained relatively constant. In comparison, cells treated with two independent EDD siRNAs (siEDD-1 and siEDD-2) exhibited dramatically increased expression levels of BUBR1, phospho-APC3, and CDC20. However, 180 min after Noc release, at a time when HeLa cells have exited mitotic arrest and begin cycling (26), the expression levels of all analyzed proteins decreased to control levels. Densitometric analysis (Fig. 4*F*) confirmed BUBR1, phospho-APC3, and CDC20 expression levels to be increased (BUBR1, >5-fold; phospho-APC3, >3-fold; CDC20, >2-fold) over control levels. Therefore, upon impairment of EDD function, mitotic cells overexpress CDC20 and phospho-APC3, two positive regulators of APC^CDC20^-associated mitotic progression (27).

##### EDD siRNA Does Not Regulate “Normal” Mitotic Duration

**FIGURE 5.**
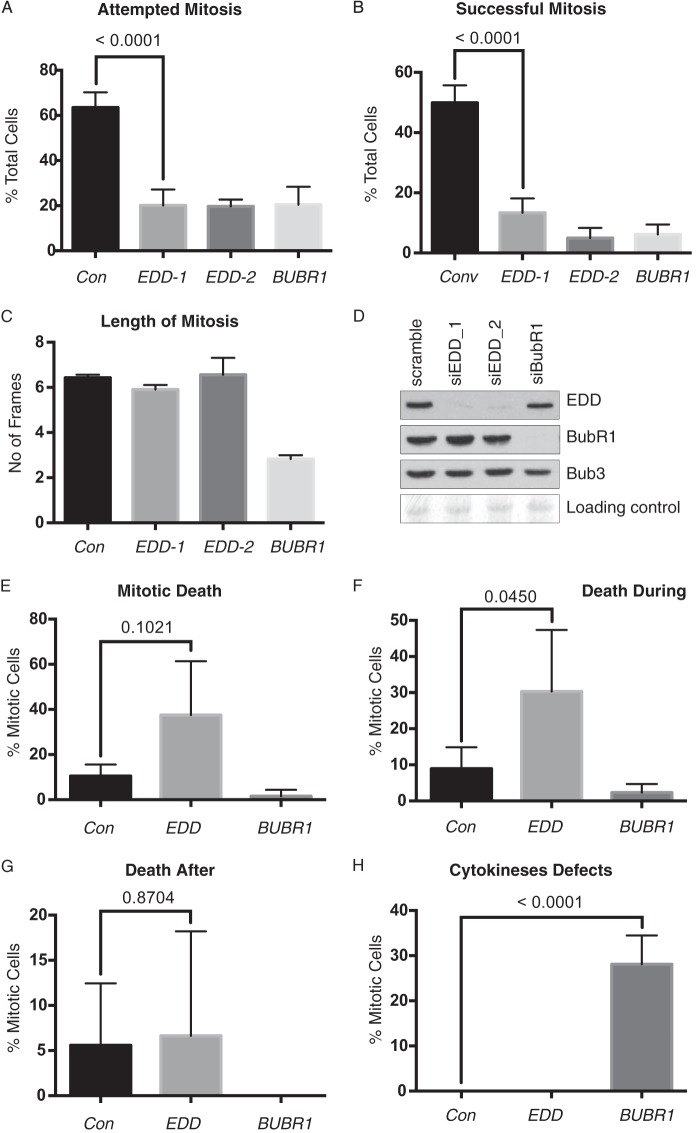
***EDD* siRNA reduces mitotic frequency and success rate, but not mitotic timing.** *A–C*, asynchronous HeLa cells were treated with *EDD*, *BUBR1*, or scrambled control (*Con*) siRNAs for 12 h prior to time-lapse microscopy. Cells were scored for their ability to initiate (*A*) and successfully complete mitosis (*B*), and we measured the duration of those successful mitoses (*C*). *D*, SDS-PAGE and Western blotting with the indicated antibodies validated the efficacy of siRNA-mediated knockdown. *E–H*, the fate of cells attempting mitosis was also assessed for overall cell death (*E*), death during mitosis (*F*), death following mitosis (*G*), and abnormal cytokinesis (*e.g.* fusion of daughter cells) (*H*). The total number of cells analyzed was >100 and, for mitotic cells, >40 for all siRNA-treated cell groups. The data in *A*, *B*, and *E–H* were analyzed by Fisher's exact test with the *p* value indicated, whereas the data in *C* were analyzed using one-way analysis of variance and Tukey's multiple comparison test, which revealed a significant difference between *BUBR1* siRNA and the other three siRNA treatments. *E–H* incorporate pooled data from *siEDD-1* and *siEDD-2. Errors bars* represent mean ± S.D.

Although all SAC components govern mitotic arrest in response to microtubule poisons, only BUBR1 and MAD2 govern the duration of normal mitoses (15), a process termed “mitotic timing.” The ability of EDD to associate with BUBR1 prompted us to address whether EDD siRNA would behave like BUBR1 and affect normal mitotic timing (15). Using live cell imaging and time-lapse microscopy of HeLa cells stably expressing RFP-tubulin GFP-histone H2B (15), we scored for successful and unsuccessful (*i.e.* dying within or soon after mitosis, exhibiting abnormal/multipolar spindles or failed/abnormal cytokinesis) mitoses and the duration of successful mitoses (see “Experimental Procedures” for details). The analysis revealed that, in comparison with scrambled siRNA treatment, *EDD* or *BUBR1* siRNA reduced both the number of attempted (Fig. 5*A*) and successful (Fig. 5*B*) mitoses, but only *BUBR1* reduced mitotic timing (Fig. 5*C*). Western blot analysis confirmed the efficient knockdown of the target proteins (Fig. 5*D*). Closer analysis of cells attempting mitosis revealed *EDD*, but not *BUBR1*, siRNA to increase cell death (Fig. 5*E*). However, statistical analysis indicated that the effects were not significant (*p* > 0.1). However, focusing on cell death events solely occurring within mitosis (Fig. 5*F*), but not after mitosis (Fig. 5*G*), revealed that *EDD* siRNA increased the frequency (*p* < 0.05). Analysis of cytokinesis also revealed that *BUBR1,* but not *EDD,* siRNA resulted in cytokinesis defects associated with incomplete cellular fission and subsequent fusion of the daughter cells (*p* < 0.001).

In conclusion, *EDD* and *BUBR1* siRNA both resulted in a significant reduction in the frequency and success rate of mitosis. However, *EDD* siRNA did not affect mitotic timing but did increase the amount of mitosis-associated cell death.

##### EDD and BUBR1 siRNA Increase γ H2AX Levels

**FIGURE 6.**
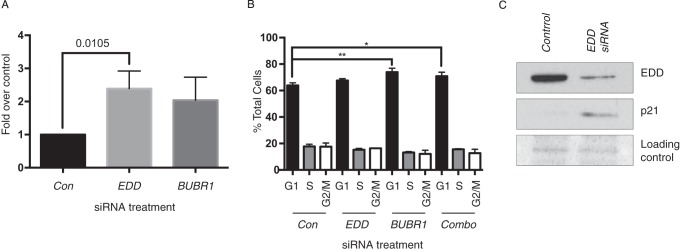
**EDD siRNA does not mediate G_1_ arrest but does increase p21 and γH2AX levels.** *A* and *B*, HeLa cells were treated with scramble control (*Con*), *EDD*, *BUBR1*, or combined (*Combo*) siRNAs for 48 h and then analyzed by γH2AX (*A*) or propidium iodide FACS analysis (*B*). *A*, values represent the -fold increase over basal γH2AX levels (47) detected in scramble control cells. *A*, data were analyzed using unpaired Student's *t* test with the *p* value indicated. *B*, data were analyzed using one-way analysis of variance and Tukey's multiple comparison test, which revealed significant differences between the control and both *BUBR1*- and *Combo* siRNA-treated samples. *A* and *B*, *errors bars* represent mean ± S.E. from three experiments. *C*, SDS-PAGE and Western blotting with the indicated antibodies confirmed the *EDD* siRNA-mediated knockdown and induction of p21.

To investigate what might be responsible for the increased cell death, we looked for signs of DNA damage. The SAC aims to delay mitotic progression to prevent improper chromosome distribution and DNA damage associated with damaged lagging chromosomes (28) and telomere exposure (29). Aberrant SAC behavior results in mitosis-associated DNA damage that subsequently leads to p53-mediated G_1_ arrest in daughter cells (30, 31). To address whether *EDD* siRNA also resulted in DNA damage, we used FACS to detect changes in the expression levels of a γH2AX, a marker of double strand breaks (Fig. 6*A*). Both *EDD* and *BUBR1* siRNA resulted in a significant increase in γH2AX levels in comparison with a scrambled control. However, FACS analysis by propidium iodide staining revealed that only *BUBR1* siRNA caused a significant increase in the G_1_ cell population in comparison with the control (Fig. 6*B*). Interestingly, Western blot analysis of *EDD* siRNA-treated cells revealed a moderate increase the expression of p21 (Fig. 6*C*), a CDK inhibitor and potent mediator of the G_1_/S checkpoint (32). Interestingly, cells transfected with both *EDD* and *BUBR1* siRNAs resulted in a FACS profile very similar to that of *BUBR1* siRNA alone (Fig. 6*C*). In conclusion, even though *EDD* siRNA increased γH2AX and p21 expression levels, it did not mediate an accumulation of G_1_ cells.

## DISCUSSION

In this study, we reveal the ability of EDD to govern the cellular response to a mitotic spindle poison (Noc), document the dynamic relationship of EDD with mitotic chromatids/chromosomes during mitosis (Fig. 2), and support the mass spectrometry-based observations that identified the association of EDD with mitotic chromosomes (33). Furthermore we also identified novel physical and genetic interactions between EDD and BUBR1 as well as the ability of EDD to suppress CDC20 expression. Taken together, our results suggest that EDD may promote SAC function in response to dramatic spindle perturbations.

Both *EDD* and *BUBR1* siRNA prevented the accumulation of G_2_-M cells in response to Noc treatment, supporting the potential role of EDD as positive regulator of a G_2_/M or M-phase checkpoint. Interestingly, combined *EDD* and *BUBR1* siRNA of Noc-treated cells revealed a more dramatic effect than single treatment alone. This observation, in combination with the ability of the two proteins to copurify, suggests that the two proteins might work together in the same pathway, although we are unable to rule out the participation of EDD in a BUBR1-independent parallel pathway.

In the presence of Noc, mitotic cells normally undergo prolonged activation of a SAC-mediated mitotic arrest caused by SAC-mediated inhibition of CDC20 function (27). Noc-treated mitotic *EDD* siRNA cells exhibited dramatically increased levels of both CDC20 and the CDC20-binding competent, phosphorylated, and active form of APC3 (Fig. 4*D*), indicative of increased APC^CDC20^ activity (25). This suggests that EDD, like BUBR1 and BUB3, normally acts to repress CDC20 function. Identification of the physical association of EDD with CDC20 and APC3 provides a potential direct molecular mechanism by which EDD, an E3 enzyme, may promote their ubiquitin-dependent degradation. CDC20, as part of the MCC, is ubiquitylated and degraded in an APC/C-dependent manner (34, 35). The ability of EDD to complex with CDC20, BUBR1, and BUB3 supports the idea that EDD could promote APC^MCC^-mediated CDC20, and potentially APC3, degradation.

The accompanying increase in BUBR1 expression upon *EDD* siRNA also suggests that EDD affects the expression of SAC components during mitosis. As with CDC20, EDD may influence APC-mediated BUBR1 ubiquitylation and degradation (36). Increased p53-mediated transcriptional up-regulation of *BUBR1* (37) could also account for the observed increase in BUBR1 expression in *EDD* siRNA cells. Our observation of *EDD* siRNA increasing the expression p21, the product of one of the target genes of p53, provides evidence for increased p53 activity in *EDD* siRNA cells. Interestingly, the increase in BUBR1 expression did not appear to correlate with increased SAC activity, as determined by the failed accumulation of a G_2_/M cell in response to Noc. Therefore, we predict that although EDD may normally suppresses the expression levels of BUBR1, it may also positively regulate the activity of BUBR1. Alternatively, EDD may only suppress the expression of certain subcellular pools of BUBR1 not incorporated into SAC or MMC complexes. Unlike BUBR1 and CDC20, BUB3 expression levels did not change through mitosis or upon *EDD/BUBR1* siRNA. A lack of rapid BUB3 protein turnover could reflect its non-catalytic scaffolding role within the SAC.

The potential ability of EDD to influence SAC and APC^CDC20/MCC^ function provides a potent means of regulating mitotic progression as well as DNA damage associated with improper/untimely mitosis (24). The increased p21 expression levels in *EDD* siRNA cells may reflect an attempted p53-mediated cell cycle arrest in response to the concomitant increase in γH2AX DNA damage signals. Supporting these observations, EDD has been proposed to be a negative regulator of p53 activity and suppressor of a G_1_ cell cycle arrest (9, 10). The absence of such an arrest in HeLa cells may potentially be explained by their expression of HPV E6, a potent inhibitor of p53 function (38).

Although *EDD* siRNA alone did not induce G_1_ arrest, in the presence of Noc, both *EDD* and *BUBR1* siRNAs caused a dramatic increase in the G_1_ cell population. Therefore, Noc treatment appeared to uncover the role of EDD in suppressing a G_1_ arrest. The ability of Noc to increase DNA damage signaling (24), particularly at telomeres (39), may provoke a robust DNA damage response (DDR) that is normally held in check by EDD. Such a role for EDD is supported by its ability to suppress DDR signaling at sites of DNA damage (40) and, specifically, at exposed telomeres (41). The role of EDD in suppressing an inappropriate DDR from exposed telomere ends may be particularly relevant because of the ability of mitotically associated Aurora-B-kinase to promote telomere-associated DDRs (29).

Previously, the SAC-regulated mitotic checkpoint and the G_1_/S DNA damage checkpoint were thought to function independently of each other. Recent evidence revealed that ataxia telangiectasia mutated (ATM), in response to exogenous DNA damage, utilizes a SAC-associated mechanism to inhibit anaphase progression (42). Therefore, ATM is capable of coordinating an interphase G_1_/S checkpoint (43) in addition to a SAC/CDC20-associated mitotic checkpoint. The ability of EDD to associate with two proteins intimately associated with ATM function (checkpoint kinase 2 (CHK2) (8) and ATM interactor (ATMIN) (44)) as well as various SAC components raises the possibility that it may act in a similar manner.

In this work, we identified the physical association of EDD with the SAC proteins BUB3 and BUBR1 and an ability to overcome Noc-mediated G_2_/M accumulation and influence the expression key mitotic regulators. Both BUBR1 and EDD are essential in early mammalian development, with homozygous *Ubr5* (45) (EDD's murine homolog) and *BUBR1* (46) null mutants exhibiting extensive apoptosis and embryonic lethality around embryonic days 8.5–9.5. These results, combined with those indicating a role in suppressing DDRs (40, 41), further implicate EDD as a potential caretaker of genomic integrity through its ability to regulate both prophase and interphase cell cycle arrests in response to cellular stress.

## Footnotes

2 The abbreviations used are:

EDD: E3 identified by differential display
SAC: spindle assembly checkpoint
APC/C: anaphase-promoting complex/cyclosome
MCC: mitotic checkpoint complex
Noc: nocodazole
IP: immunoprecipitation
Tax: Taxol
DDR: DNA damage response.
